# Citation Analysis of Hepatitis Monthly by Journal Citation Report (ISI), Google Scholar, and Scopus

**DOI:** 10.5812/hepatmon.7441

**Published:** 2012-09-07

**Authors:** Seyyed Mohammad Miri, Azam Raoofi, Zahra Heidari

**Affiliations:** 1 Baqiyatallah Research Center for Gastroenterology and Liver Diseases, Tehran, IR Iran; 2 Kowsar, Hoensbroek, The Netherlands; 3 Department of Clinical Psychology, Shahid Beheshti University of Medical Science and Health Services, Tehran, IR Iran

**Keywords:** Citation Analysis, Impact Factor, Hepatitis Monthly, ISI, Scopus, Google Scholar

## Abstract

**Background:**

Citation analysis as one of the most widely used methods of bibliometrics can be used for computing the various impact measures for scholars based on data from citation databases. Journal Citation Reports (JCR) from Thomson Reuters provides annual report in the form of impact factor (IF) for each journal.

**Objectives:**

We aimed to evaluate the citation parameters of Hepatitis Monthly by JCR in 2010 and compare them with GS and Sc.

**Materials and Methods:**

All articles of Hepat Mon published in 2009 and 2008 which had been cited in 2010 in three databases including WoS, Sc and GS gathered in a spreadsheet. The IFs were manually calculated.

**Results:**

Among the 104 total published articles the accuracy rates of GS and Sc in recording the total number of articles was 96% and 87.5%. There was a difference between IFs among the three databases (0.793 in ISI [Institute for Scientific Information], 0.945 in Sc and 0.85 GS). The missing rate of citations in ISI was 4% totally. Original articles were the main cited types, whereas, guidelines and clinical challenges were the least ones.

**Conclusions:**

None of the three databases succeed to record all articles published in the journal. Despite high sensitivity of GS comparing to Sc, it cannot be a reliable source for indexing since GS has lack of screening in the data collection and low specificity. Using an average of three IFs is suggested to find the correct IF. Editors should be more aware on the role of original articles in increasing IF and the potential efficacy of review articles in long term impact factor.

## 1. Background

The journal of “Hepatitis Monthly” (Hepat Mon) as one of the leading journals in the field of hepatology in the region has been published since 2001. The Journal context is devoted to particular compilation of the latest worldwide and interdisciplinary approach and findings including original manuscripts, meta-analyses, review articles, health economic papers, debates and consensus statements of clinical relevance of hepatological field especially liver diseases. By the end of 2010, more than 220 papers have been published in 28 issues from them 210 articles are indexed in ISI (Institute for Scientific Information) Web of Science since 2007. Full-text of articles can be found in many indexing sources like Scopus and EMBASE which allows the wide exposure and opportunity for sound and insightful content to be cited by other researchers throughout the world. Citation analysis, as the examination of the frequency, patterns, and graphs of citations in articles and books, is one of the most widely used methods of bibliometrics and is suitable application for the identification of expert referees to review papers, to provide transparent data in support of academic merit review, tenure, and promotion decisions. The citation analysis tools can be used for computing the various impact measures for scholars based on data from citation indices.

Web of Science (WoS) is an online academic citation indexing source provided by Thomson Reuters since 1900. Journal Citation Reports (JCR) is a comprehensive resource that allows readers to evaluate and compare journals using citation data drawn from over 11,000 scholarly and technical journals of more than 3,300 publishers in over 80 countries. JCR shows the most frequently cited journals and the highest impact factor (IF) of the journals in a field. Google Scholar (GS) is a freely accessible web search engine that indexes the full text of scholarly literature across an array of scholarly publishing formats either from peer-reviewed or non-peer-reviewed journals since 2004. Scopus (Sc), officially named SciVerse Scopus, is a bibliographic database containing abstracts and citations for academic journal articles which covers nearly 18,000 titles from over 5,000 international publishers, including coverage of 16,500 peer-reviewed journals in the scientific, technical, medical, and social sciences (including arts and humanities). It is owned by Elsevier and is available online by subscription.

## 2. Objectives

We aimed to evaluate the accuracy of Hepatitis Monthly IF presented by JCR in 2010 comparing with GS and Sc and evaluate the advantages and threats of JCR in comparison with GS and Sc.

## 3. Materials and Methods

Based on JCR 2010, we selected all articles of Hepat Mon published in 2009 and 2008 which had been cited in 2010. Using JCR 2010, Sc and GS report, we gathered all data in a spreadsheet then cleared dataset and prepared them for analysis. Calculation of the IF was done based on the JCR method provided by Thomson Reuters (Table 1). The IF from JCR was assumed as the gold standard when calculating others from Sc and GS.

**Table 1:** Method of Calculating Impact Factor of Hepatitis Monthly in 2010 Based on JCR

	**2009**	**2008**	**Sum**
Citations in 2010 to articles published in	A	B	A+B
Number of articles published in	C	D	C+D
Calculation of Impact Factor			(A+B) / (C+D)

### 3.1. Method of data collection and analysis

For the citation indicator of Hepatitis Monthly, the published articles during 2008 and 2009 have been considered for searching in three databases: ISI, Sc, and GS. Each article searched separately in the databases and all citations recorded in a spreadsheet. Afterwards IFs were manually calculated from the spreadsheet which can be found in Table 2. To calculate IF of Hepat Mon in WoS, it was searched through the Cited Reference Search in WoS as well as the final report of JCR, 2010. According to its policy, ISI don’t count the letters to the editors and editorial articles in IF calculation. Notably ISI was considered as the gold standard for calculations.

**Table 2:** Total Number of Articles, Citations and Self-Citations of Two Volumes (2008 and 2009) of Hepatitis Monthly in Google Scholar, Scopus and ISI

**Vol ** **(issue)**	**Published Articles in Hepat Mon, No.**	**Indexed Articles, No. **	**Total Citations, No.**	**Self-Citations, No.**	**Self-Citation Rate, %**	**Impact Factor**	**Impact Factor Without Self-Citation**
		**Google Scholar**			29.41	85/100 = 0.85	0.600
8(1)	13	12	9	3			
8(2)	13	11	8	0			
8(3)	13	13	10	2			
8(4)	13	13	18	8			
9(1)	13	13	10	2			
9(2)	13	13	14	4			
9(3)	13	12	13	5			
9(4)	13	13	3	1			
Total	104	100	85	25			
		**Scopus**			29.06	86/91 = 0.945	0.670
8(1)	13	12	10	3			
8(2)	13	13	10	0			
8(3)	13	10	8	2			
8(4)	13	11	16	8			
9(1)	13	11	10	2			
9(2)	13	11	14	4			
9(3)	13	13	16	5			
9(4)	13	10	2	1			
Total	104	91	86	25			
		**ISI ^a^**			30.43	69/87 = 0.793	0.551
8(1)	13	11	10	3			
8(2)	13	11	5	0			
8(3)	13	10	3	1			
8(4)	13	11	15	8			
9(1)	13	12	8	2			
9(2)	13	11	13	2			
9(3)	13	11	16	5			
9(4)	13	10	2	0			
Total	104	87	69 ^b^	21			

^a^The results in this section is based on JCR

^b^The actual citation number based on web of science were 71 citations (missing rate in comparison with JCR calculation: 4%)

## 4. Results

Among 104 total published articles in Hepatitis Monthly during 2008 and 2009, 87, 100, and 91 articles were recorded in ISI, GS, SC respectively (Table 2). Due to omission of “letters to editor” and “editorials” in calculation of IFs of ISI, the real number of published articles were different from those indexed in ISI. Consequently and thanks to assumption of ISI as the gold standard, the accuracy rates of GS and Sc in recording the total number of articles were 96% and 87.5%, respectively. There was a difference between IF among three databases. As shown in Table 2 the IF of Hepat Mon in 2010 was 0.793 in ISI, while it was 0.945 and 0.85 in SC and GS. There were 72 citations in ISI in 2010 to the published articles in 2008 and 2009, while according to JCR, total number of citations in 2010 to the published articles in the same year (2008 and 2009) were 69. Therefore, the missing rate of citations in ISI was 4% totally (Table 2).

**Figure 1:**
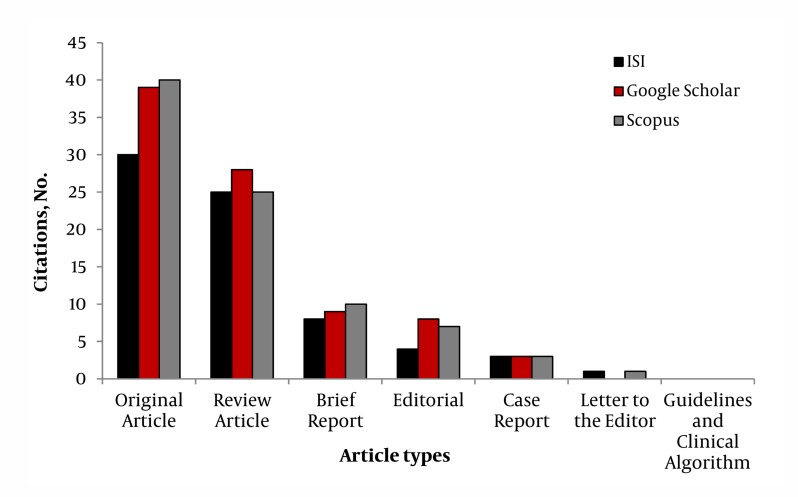
Comparison of Number of Citations in ISI, GS, and Sc Based on Article Types Published in Hepatitis Monthly (2008, 2009)

Among all published articles, two systematic reviews from Iran had the most citations which one of them was published in 2008 (n = 9) and another in 2009 (n = 7). Even though, there was a difference between the calculated IF in GS and Sc with ISI, Table 3 demonstrated that the mentioned difference was shown only in a few articles. The main discrepancy was in one original article published in 2009. According to data from GS this article is cited 5 times comparing to ISI and Sc which was two times. Figure 1 shows that among articles published in Hepat Mon during 2008 and 2009, original articles were the main cited types while guidelines and clinical challenges were the least types. Whereas if we consider proportion of citations to the types of articles, review articles have the most citation. Furthermore, case reports were the only type of articles which were the same in citation numbers in all the databases (Figure 1). No significant difference was detected in all other types of articles except original articles which represented a significant difference between ISI and the two other databases.

**Table 3:** Comparison of Citations to the Top 20 Articles of Hepatitis Monthly in ISI, GS and Sc During 2008-9

**Article Title**	**Article Type**	**Volume (Issue)**	**Citation in ISI, No.**^a^	**Citation in GS, No.** ^a^	**Citation in Sc, No.** ^a^
Hepatitis B Virus Infection in Iran: A Systematic Review	Review Article	8(4)	9	9	9
Hepatitis C Infection in the General Population of Iran: A Systematic Review	Review Article	9(3)	7	7	7
Hepatitis C Virus Genotype Distribution in Shiraz, Southern Iran	Original Article	9(2)	3	3	3
Epidemiology of Chronic Hepatitis C Virus Infection in High Risk Groups	Original Article	8(1)	2	1	1
Study of Admission Rate of Hepatitis B Surface Antigen Positive Patients in 50 Dentistry Centers in Tehran (Spring 2003)	Brief Report	8(1)	2	2	2
Potential Activity of Camel Milk-Amylase and Lactoferrin against Hepatitis C Virus Infectivity in HepG2 and Lymphocytes	Original Article	8(2)	2	2	2
The Emerging Extrahepatic Manifestations of Hepatitis C Virus Infection in Chronic Hepatitis and Mixed Cryoglobulinemia	Review Article	8(3)	2	3	2
We Have More Data Regarding Epidemiology of Hepatitis D in Iran but There are Defects to be Filled Yet!	Editorial	8(4)	2	2	2
Meningoencephalitis of Hepatitis A in Adult Man: A Case Report	Case Report	8(4)	2	2	2
Comparison of Seroepidemiology and Transmission Modes of Viral Hepatitis C in Iran and Pakistan	Review Article	9(1)	2	3	3
Hepatitis B Markers in Isfahan, Central Iran: A Population-Based Study	Original Article	9(1)	2	5	2
Trends in Seroprevalence of Hepatitis B, Hepatitis C, HIV, and Syphilis Infections in Iranian Blood Donors from 2003 to 2005	Original Article	9(1)	2	2	3
Prevalence of Hepatitis B and C Infection in Hemodialysis Patients of Rasht (Center of Guilan Province, Northern Part of Iran)	Original Article	9(1)	2	2	3
Distribution of Hepatitis C Virus Genotypes in Iran: A Population-Based Study	Original Article	9(2)	2	1	2
Thyroid Dysfunction in Patients with Chronic Viral Hepatitis B and C during Alpha Interferon Therapy	Original Article	9(2)	2	2	2
Seroprevalence of Hepatitis B Virus among Pregnant Women in Northern Turkey	Brief Report	9(2)	2	1	2
Mass Vaccination Campaign against Hepatitis B in Adolescents in Iran: Estimating Coverage using Administrative Data	Original Article	9(3)	2	2	2
Hepatitis B Infection in Hemodialysis Patients in Tehran Province, Iran	Original Article	9(3)	2	2	2
Seroprotection of Hepatitis B Vaccine and Need for Booster Dose: A Meta-Analysis	Review Article	9(4)	2	2	1
Hepatitis D Is a Forgotten Problem in Hemodialysis Patients in the World	Editorial	8(1)	1	0	0

^a^Abbreviations: GS, Google Scholar; ISI, Institute for Scientific Information; Sc, Scopus

Based on JCR, out of all citations to Hepatitis Monthly in 2010, “IRAN RED CRESCENT MED” had the most citations (n = 24) and then “ASIAN PAC J CANCER P” (n = 5) and “ARCH MED RES” (n = 4) were in the second and third levels (Table 4). Moreover, the most citations to Hepat Mon were from Iran (15.78%), Japan (3.28%) and USA (2.63%). Analyzing IF of the journals that cited to Hepat Mon doesn’t show any relationship between the journals IF and number of citation to Hepat Mon (the average IF of 1.86 for the first 10 journals compare to 2.139 for the second 10 journals). Among all citations in Hepat Mon, 90 citations belonged to “HEPATOLOGY” (IF: 10.885), 50 to “J HEPATOL” (IF = 9.334), 36 to “GASTROENTEROLOGY” (IF = 12.032), and 36 to “NEW ENGL J MED” (IF = 53.486). Therefore, there was no correlation between IF and number of citations in Hepat Mon (Table 5). Generally, the average IF of the first 10 journals is 10.517 and the average IF for the second 10 journals is 9.05.

**Table 4:** Distribution of the Top 20 Journals Which Cited in 2010 to Published Articles of Hepatitis Monthly (Data Gathered From JCR, ISI, 2010)

**Impact Factor**	**Citing Journals **	**Countries**	**All Years **	**2010**	**2009**	**2008**	**2007**
-	All Journals	-	152	24	32	37	29
0.36	Iran Red Crescent Med	Iran	24	3	8	6	4
1.24	Asian Pac J Cancer P	Japan	5	0	1	1	1
1.986	Arch Med Res	USA	4	1	2	0	1
2.24	World J Gastroentero	China	3	0	0	2	0
1.093	Hemodial Int	USA	3	0	0	2	0
1.635	J Public Health Pol	England	3	0	1	1	1
3.502	J Viral Hepatitis	England	3	0	0	2	0
2.546	Virol J	England	3	0	2	0	0
3.84	Liver Int	Denmark	2	1	0	0	0
0.166	Pak J Med Sci	Pakistan	2	0	0	0	0
1.098	Ther Apher Dial	Australia	2	1	1	0	0
2.658	Am J Nephrol	Switzerland	2	0	1	0	1
2.551	Clin Ther	USA	2	0	0	0	1
6.186	Cochrane DB Syst Rev	England	2	0	2	0	0
-	Cienc Saude Coletiva	Brazil	1	0	0	0	1
1.75	Am J Ind Med	USA	1	0	0	1	0
3.841	Anal Bioanal Chem	Germany	1	0	0	0	1
1.867	Ann Hepatol	Mexico	1	0	0	1	0
1.199	Arch Med Sci	Poland	1	0	0	1	1.199
0.248	Asian Biomed	Thailand	1	0	0	1	0.248

**Table 5:** Distribution of the Top 20 Journals Which Are Cited in Hepatitis Monthly in 2010 (Data Gathered From JCR, ISI, 2010)

**Impact Factor**	**Cited Journal **	**All Years**	**2010**	**2009**	**2008**	**2007**
10.885	Hepatology	90	5	7	5	15
9.334	J Hepatol	50	0	5	4	1
12.032	Gastroenterology	36	10	0	2	2
53.486	New Engl J Med	36	0	8	0	2
2.895	J Med Virol	33	1	2	4	0
3.502	J Viral Hepatitis	31	4	4	1	6
2.24	World J Gastroentero	31	0	3	4	6
6.882	Am J Gastroenterol	30	0	1	0	0
0.342	Jcpsp-J Coll Physici	20	0	1	2	2
3.572	Vaccine	19	0	1	0	3
2.41	J Gastroen Hepatol	19	0	3	2	1
33.633	Lancet	16	0	2	0	0
1.348	Arch Iran Med	14	0	0	1	1
2.06	Digest Dis Sci	13	0	0	3	2
10.614	Gut	12	0	0	0	0
-	J Pak Med Assoc	12	0	0	1	0
16.729	Ann Intern Med	11	0	0	0	0
5.189	J Virol	10	0	0	0	0
3.564	Nephrol Dial Transpl	9	0	0	1	0
1.756	Intervirology	9	1	1	3	0

## 5. Discussion

The accuracy rates of GS and Sc in recording the total number of articles were 96% and 87.5%. Although GS had indexed more articles than Sc, it is not reasonable to rely on it for counting the total number of articles since fundamentally GS is a robot which collects data via the websites with neither human interaction nor data cleaning while Sc is guided and supervised by operators. by another meaning, GS has high sensitivity and low specificity vs. Sc with high specificity, while GS reports all citations from different types of publications, therefore, it cannot be as specific as the two other databases (1). GS have found to be sometimes inadequate, and less often updated (2). Moreover, Using GS does not guarantee to access the full-text availability for the readers since it is regulated via the journal’s publisher (3). Another important factor is that the relative coverage of GS varies by discipline compared to other general databases (4). It is also indicated that reliability of each database depends on some factors such as year and the subject of publication (5). Despite much more reliability and validity of Sc, there are some doubts about Sc. Since the Elsevier is the owner of Sc, and is also one of the main international publishers of scientific journals, some users have misgivings about a potential conflict of interest in the choice of the periodicals to be included in the database (3).

There was a difference between IF among three databases. (GS = 0.85, Sc = 0.945). Notably Sc covers more international and open access journals than the other databases (5) and this could be the potential reason for higher IF among all. Although we found difference among those three databases, Bauer et al. in 2005 showed no significant difference between results by Sc and WoS (1). Just similar to Bauer (1), Bakkalbasi et al. in 2006 reported differences in citations of all pairs databases except GS and Sc (5). In contrast with previous studies by Bauer and Bakkalbasi and like to ours, another study revealed higher coverage of articles by Sc than WoS (6). “Cited by” feature in GS, found to be inadequate and less often updated in comparison to the other two databases (2). Respect to the nature of GS which is a crawler, a significant problem with GS is the secrecy about its coverage because some publishers do not allow it to crawl their journals. For instance Elsevier journals were not included before mid-2007, when Elsevier began to make most of its ScienceDirect content available to GS and Google's web search (1, 7). List of journals crawled are not known in GS as well as the frequency of its updates (2). Copyright issues in accessing to the contents of the most expensive commercial databases are still discussed between Google and the publishers like Thomson Rheuters. GS crawlers collect blindly citations and manipulated hitting in a webpage raises its ranking consequently brings the page to the tops of the search results. This false effect of GS on citation counts is criticized with many searchers. Therefore citation counts from GS are not a reliable metrics for counting the h-index and impact factor.

Another issue for searchers in GS is searching with Interpunctuation characters in titles which produce wrong search results, and authors are assigned to wrong papers, which leads to erroneous additional search results. Some editors prefer to negotiate with ISI to recategories the published articles to be considered as citable articles (8). Bagatin et al. believe IF cannot completely represent quality of articles, since review articles have a high potential for citations while the publishing case reports usually have a negative effect on citation on account of few citations (9). Based on the types of articles published in Hepat Mon during 2008 and 2009, no significant difference was detected in all other types of articles except original articles which represented a significant difference between ISI and the two other databases. Review articles receive extremely high citations and review journals have some of the highest impact factors (10). There is a skewed distribution of citations in most fields. The so-called 80/20 phenomenon applies, in that 20% of articles may account for 80% of the citations (11). Publishing mediocre review papers will not necessarily boost a journal’s impact (11).

As the free access of PubMed and GS comparing with WoS and Sc absorbs more readers and citations while Sc offers about 20% more coverage than WoS, whereas GS offers results of inconsistent accuracy. Till now PubMed remains an optimal tool in biomedical electronic research. Currently Sc is limited to the recent articles that have been published since 1995 following which it cannot overcome WoS, in spite of wider journal coverage (2). However, some studies emphasize that Sc is more user friendly with shorter time span (12). Thanks to alerting features via Sc which allows authors to track all published articles, it also offers authors a profile which covers affiliations, number of publications and their bibliographic data, references, and details on the number of citations each published document has received. H-index then will be calculated in each profile (12). Garfield believes that 24% of references contain only 25 journals (little more than 1 percent of SCI coverage) (13). This analysis gives good reason for concern about any increase in the number of scientific and technical journals. Increased number of journals makes coverage of the literature more difficult, but it seems that most of the current journals play only a marginal role, if any, in the effective transfer of scientific information (13). Of 2200 journals covered by the SCI in 1969, 500 journals publish about 70% of all published articles (13).

None of the three databases succeed to record all articles published in the journal. Despite high sensitivity of GS comparing to Sc, it cannot be a reliable source for indexing since GS has lack of screening in the data collection and low specificity. According to the highest IF in Sc comparing to GS and ISI it is suggested using an average of three IFs to find the correct one. Editors should be more aware on the role of original articles in increasing IF and the potential efficacy of review articles in long term impact factor.
